# How Bees Discriminate a Pattern of Two Colours from Its Mirror Image

**DOI:** 10.1371/journal.pone.0116224

**Published:** 2015-01-24

**Authors:** Adrian Horridge

**Affiliations:** Australian National University, Canberra, Australia; University of Melbourne, AUSTRALIA

## Abstract

A century ago, in his study of colour vision in the honeybee (*Apis mellifera*), Karl von Frisch showed that bees distinguish between a disc that is half yellow, half blue, and a mirror image of the same. Although his inference of colour vision in this example has been accepted, some discrepancies have prompted a new investigation of the detection of polarity in coloured patterns. In new experiments, bees restricted to their blue and green receptors by exclusion of ultraviolet could learn patterns of this type if they displayed a difference in green contrast between the two colours. Patterns with no green contrast required an additional vertical black line as a landmark. Tests of the trained bees revealed that they had learned two inputs; a measure and the retinotopic position of blue with large field tonic detectors, and the measure and position of a vertical edge or line with small-field phasic green detectors. The angle between these two was measured. This simple combination was detected wherever it occurred in many patterns, fitting the definition of an algorithm, which is defined as a method of processing data. As long as they excited blue receptors, colours could be any colour to human eyes, even white. The blue area cue could be separated from the green receptor modulation by as much as 50°. When some blue content was not available, the bees learned two measures of the modulation of the green receptors at widely separated vertical edges, and the angle between them. There was no evidence that the bees reconstructed the lay-out of the pattern or detected a tonic input to the green receptors.

## INTRODUCTION

The study of bee colour vision has attracted many naturalists over the past centuries, but has not revealed the kind of mechanism involved. In the 19^th^ century, some critical observers found that blue was the preferred colour, and that bees were attracted to landmarks, not to the colours of flowers [1–3]. Karl von Frisch showed that bees trained to go to a coloured target for a reward of sugar would then distinguish that colour (except bluish-green or grey) from all shades of grey [4]. These results provided support for the popular belief that bees have colour vision, but this conclusion was seriously challenged. From extensive testing of patterns behind glass to eliminate effects of odours, Hess [5] found that bees trained to go to yellow could not distinguish yellow from other colours, but he was ignored and soon died. The belief that bees have colour vision prevailed, and became accepted as trichromatic colour vision [6].

**Fig 1:**
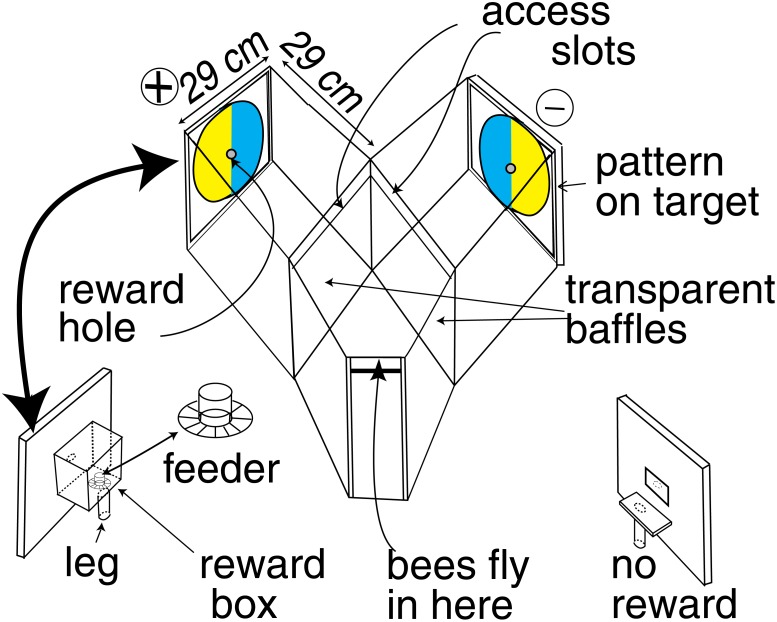
The Y-choice apparatus. The bees entered through the horizontal slot at the front into a choice chamber from which they could see both targets. They selected one pattern and were scored as they passed over one of the transparent baffles through the narrow horizontal slot to reach the reward hole. When satisfied at the feeder, they exited by the way they came. To make the bees look at the patterns and learn, the reward with its pattern changed sides every 5 or 10 min.

**Fig 2:**
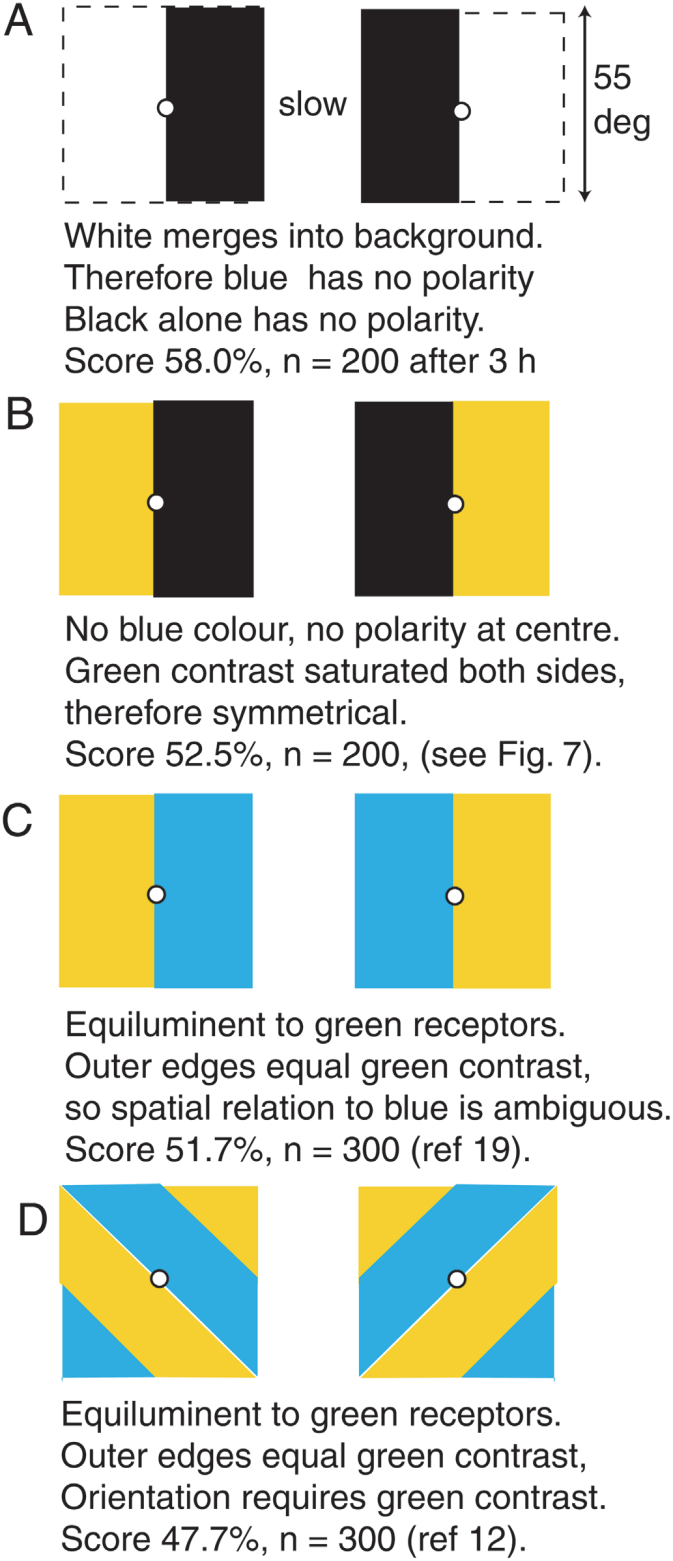
Patterns that the bees could distinguish only after long training or not at all. Possible explanations for the failures are given below each pair. Pairs B, C, and D were discriminated when an additional black vertical line was added.

There were problems with the early experimental demonstrations. Colour-blind people can also distinguish between flowers of different colours, and von Frisch’s demonstration of discrimination of patterns with two colours in different positions (Figs. 1 and 2) does not require colour vision. Furthermore, bees cannot discriminate some patterns of two colours at all (Fig. 2) [7, 8].

In early work, Mathilde Hertz [9] found that bees discriminated between black/white patterns by the total length of edge, which she called ‘figural intensity’, and later work by others showed that bees detected the location, average orientation and length of edges irrespective of pattern [10]. Figural intensity was later recognized as ‘receptor modulation’, and can be approximated as (contrast × length of edge) [11, 12]. With reference to colour, recent work showed that contrast above 30% was saturated, and the discrimination of orientation was done solely by the green receptor channel [13]. In the absence of green contrast, vertical and horizontal gratings were discriminated by measuring the modulation of blue receptors [8]. In the discrimination of black patterns from white ones, bees measured and compared the content of blue and the amount and positions of green modulation, with no suggestion of the use of a tonic green receptor input [12]. The amount of blue and the modulation of each type of receptor were therefore probable inputs for the discrimination of colour.

In the neuron anatomy, recently, it was confirmed that green receptor axons of the bee all terminate at the lamina [14], making it unlikely that green tonic receptor responses are transmitted beyond there. The blue receptor axons run straight through the lamina to the medulla [15] so that blue content is retained.

Electrophysiological recordings from the optic lobe show that the tonic and phasic responses of the photoreceptors are processed separately as far as the lobula, and that most neuron responses are phasic [16]. The tonic blue-sensitive neurons of the deep optic lobe of the bee have very large fields, ranging across the whole eye [16] so that they would not resolve patterns. Furthermore, the rapid changes of sensitivity over a 1000-fold range, and the scanning motion of the bee in flight make it unlikely that the recognition of colours depends on the ratios of the photon fluxes.

In the early 1990’s it was realised that the cues that the bees used to distinguish patterns could be inferred from failures of trained bees to distinguish between carefully selected test patterns [17]. When the bees failed in the tests, the features they had detected and learned from the training patterns were missing. As corroboration, in other tests the missing features were replaced and the bees were successful. This strategy led to the discovery that the green and the blue feature detectors of edges were only 3° long [18]. When ultraviolet was excluded, only a few inputs were available in black/white patterns [10]. These inputs were:- modulation of the green [19] and the blue [8, 20] receptors, local averaged edge orientation via the green receptor pathway [8, 13], positions of the hubs of radial and tangential arrangements of edges [10], and amount of blue (solid angle times intensity) in large fields [12]. Only one input of each type was learned in each separate task [21]. These inputs were retinotopic, meaning they were learned and recognized at the place on the eye where they were detected, and were measured quantitatively [12, 17]. Pattern or shape vision was ruled out because edge orientation was averaged over local regions of the eye [10]. The belief that bees could recognize global features of whole patterns, and could generalize between similar patterns, was replaced by firm observations that they detected cues, not patterns [10]. They generalized between similar black/white patterns because they had available only the limited repertoire of these six different inputs. Generalization was not a clever performance, it was confusion caused by an insufficient variety of cues to distinguish every pattern [22].

### The problem with reference to colour

Almost all past observations of bees’ responses to coloured patterns or flowers were descriptions or measurements of performance, and never opened up the analysis of mechanisms. The colour triangle [23] invented by Maxwell for human vision was a summary of the calculated ratios of the inputs to the receptors, telling nothing about subsequent processing, and has never been located in any animal.

The first requirement was to discover the kind of system involved in the bees learning, memory, and recognition of colour. For example, maybe it was continuously impressionable like wax, or maybe a small variety of discrete detectors were triggered by anticipated features. A second requirement was to discover what bees measured in colours. Detection of colour could depend on phasic as well as tonic responses of the green or the blue receptors, so that four independently variable inputs were available. By ‘tonic’, I mean maintained responses to photon flux, and by ‘phasic’ I mean the modulation of receptor responses as bees in flight scan across edges.

## METHOD OF TRAINING AND TESTING

The bees (*Apis mellifera*) were from a single isolated hive about 30 m away from the experiments. The workers had identical genetic code and similar previous experience. After the majority of the competing spring blossom had finished, a few bees were first bought into the training apparatus by placing a feeder (Fig. 1) with strong sugar solution (20% w/w) near to the hive, then moving the feeder, with a few bees, step by step into the apparatus. There they had a choice of two further chambers; effectively, a very short Y maze leading into a reward chamber [7, 8, 10, 13]. These pioneers attracted a few recruits to the strong sugar and some bees returned on the next day. The solution strength was then reduced until recruits were no longer attracted, and a large second feeder with dilute (3% w/w) sugar solution was prominently placed about 5 m from the apparatus. This is a decoy for the unwanted recruits and scout bees, so that they fail to find the small entrance to the apparatus. It took several days for the number of experimental bees to settle down to a small group of 12–15 bees that arrived regularly. To keep the bees coming without attracting recruits, the strength of the sugar solution was adjusted to the temperature each day. Recruited bees were excluded or ignored. Training and marking the selected group preferably began each Monday morning with tests and continued training all week. Each bee was uniquely marked with a coloured spot on the abdomen and thorax. An experiment usually lasted all week or more. One bee at a time was allowed into the apparatus.

The whole study of colour occupied parts of three years, and the apparatus and procedure was never changed since 1995 [7, 8, 10–12]. The bees had a choice between two training targets, each displaying a pattern subtending not more than 55° at a range of 29 cm. The central hole in one target, labeled positive in all the illustrations, led to a reward of sugar solution while the hole in the other led nowhere. The bees were obliged to make their choice from behind a transparent baffle that made them pause in flight and look from a known distance at the patterns displayed on the targets (Fig. 1). Both targets could be seen from the choice chamber. The targets changed sides every 5 min during training so the bees could learn only the displayed patterns to identify which side to go for the reward. This alternation of the two sides eliminated or reduced errors due to side preferences of the bees, odours, uneven lighting, or other distractions in the apparatus. In the tests, bias was cancelled by reversing the sides of the patterns in alternate tests. Occasionally, when a side preference was suspected, the bees were tested with identical clean new patterns on the two targets and by placing the reward on both sides. Each bee passed over a baffle to reach a pattern, and exited by the same route.

Ultra-violet reflections were reduced by working away from direct sunlight, by the polycarbonate sheet forming the roof of the apparatus, and by use of white or coloured papers that reflected negligible UV. The coloured papers in the displays were from the numbered commercial series, manufactured by Canson or by Spektrum and available world-wide. To avoid ambiguity, the names of the colours in this study were the names of the commercial papers, as sold. White was computer printer paper made from wood pulp, which also reflected negligible UV. For the relative strengths of the inputs from each coloured paper, refer to Table 1. The spectral calibrations and the methods of obtaining them are published in several previous works [7, 8, 13, 19].

**Table 1:** Relative receptor excitations by the different papers relative to the white paper (100%), and contrasts between some pairs of papers.

**Name of paper color**	**Blue receptor**	**Green receptor**
	*Canson papers*	
Hemp 374	34.2	56.3
Ultramarine 590	33.8	20.7
Billiards green 576	17.0	22.3
Buff 384	25.7	41.7
Blue 395	54.2	40.0
White paper	100	100
Contrast 374/590	**0.06**	0.46
Contrast 384/595	0.36	**0.02**
	*Spektrum papers*	
Blue 57	33.8	21.4
Green 63	12.0	24.5
Yellow 8	13.1	78.1
Contrast 57/63	0.58	**0.08**
Contrast 63/8	**0.05**	0.63
Contrast 57/8	0.53	0.68

Equiluminant pairs are shown in bold print. The names of the colours are those used by the manufacturers. The responses of the bees to contrast saturated near values of 0.4 in bright light. The Canson papers were kindly calibrated by M. V. Srinivasan and S. W. Zhang. For Spektrum colours, see [13].

Working with a single bee is possible but tedious and extremely slow, so a small group of bees from the same hive were trained together. A short experiment can then be completed in a week, but a few problems arise. When one or two bees go missing during the experiment they can be replaced by recruits that learn to a high score in two to three hours. Some bees prefer to work in the early morning but fail to appear in the afternoons; occasionally a bee never learned to a high score, but was always left to make a contribution. All small departures from homogeneity of the samples increased the standard deviation a little, but that did not matter when all that was required was a pass or fail.

Each Monday morning a new group of 12–15 marked bees were trained over a period of 2–3 h to a sufficient level, usually over 80% correct, to give clear positive or negative answers in the tests and to avoid trends caused by further learning during the experiment. They were scored as they passed a baffle. Each 5 min test was followed by 15–20 min of continued training then a test with the patterns on the other side. Testing at intervals of 30–50 min and re-training between tests usually lasted all week. Several different tests were given before the same test was displayed again, to prevent the bees from learning from any one repeated test.

The success of the experiments depended very much on first identifying a set of effective training patterns. By use of training patterns equiluminant for the blue or the green receptors, it was possible to train bees via the green receptor or the blue receptor pathway alone, or by both. They could be trained to measure the input by training on two gratings of similar colour but differing period. Bees ignored cues that were the same on both targets because they learned on one and unlearned on the other. Successful discriminations of two different colours provided no information about the mechanism. It was essential to give a succession of different tests, so that they could not learn any one test. Therefore it was convenient to give a variety of tests that supported each other.

The main effort was to determine whether trained bees passed or failed each test. From the result of all the tests in each experiment, it was possible to deduce exactly what the bees had detected, learned and later recognized from that pair of training patterns. In contrast to the popular belief that they learned when rewarded, the bees usually learned the unrewarded target first, because learning was by trial and error, and they recognized their error when not rewarded.

### Statistics

Scores at each test are presented as the percentage of correct choices and the number of choices by the group of 12–15 trained bees. It is important to understand that the result of each test is a unique piece of data for that pair of test patterns, and unrelated to the other tests in that experiment. The test scores were not comparable to other tests because each is a forced choice between two unfamiliar targets, so in an ideal world, the bees would be 50% or 100% correct, i.e., fail or pass. Therefore, in each test, only a significant pass or fail was required, so we need to know whether each test score was theoretically different from 50%. Every effort was made to design test patterns that gave clear pass/fail answers.

**Fig 3:**
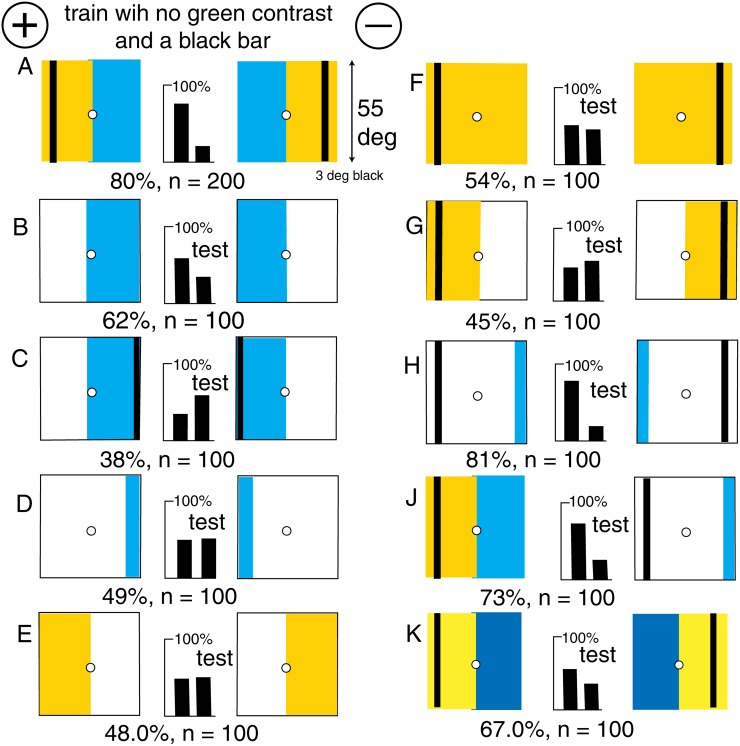
A black line enabled bees to distinguish patterns equiluminant for green receptors. (A) The training patterns. (B) The trained bees discriminated the blue panels alone. (C) Moving the black line to the outer edge of the blue reversed the preference (p < 0.02). (D, E, F, G) Narrow blue bars, buff panels alone, a black line on a buff target, or on a buff panel, were not discriminated. (H) A black line and a blue bar, forming a skeleton pattern, were as good as the training pattern. (J) The training pattern was easily distinguished from the skeleton pattern. (K) Changing the blue contrast at the central midline to green contrast had little effect because the black line already displayed strong green contrast. For scores of 62 or more, or 38 or less, and n = 100, p < 0.02 (2%), which was acceptable.

With continued training and other tests intervening, each test was continued until 100–200 counts had been made. Because the data are frequencies, standard deviations were calculated from the formula s. d. = √[p.(1-p)/n] where p is the measure of probability of a correct response, and n is the number of observations (24). This formula is valid when the choices of the bees are independent and the scores have no trend. As a quick rule of thumb, a score of more than 0. 57 (57%) for n = 200, or a score of 0.60 (60%) for n = 100, was then more than two standard deviations greater than chance (p < 0.05) which was acceptable. In Fig. 3B, for example, the score was 0.62 (62%), n = 100.

In the more exact method, we wish to reject the null hypothesis that the observed score is not significantly different from 50%, in a random sample from a binomial distribution. An exact p value was computed using a binomial test, without any normal assumption or central limit theorem claim, with an estimate of the bias and a two-sided test to reject the hypothesis that the bias is > 0.5. The test was two sided because values on either side of 0.5 occurred. In Fig. 3B, we have n = 62%, so P(estimated bias - 0.5) > 0.12 where the probability is taken with respect to a binomial distribution with bias 0.5 and n = 100. From a table of p values, p = 0.020, (2%), which is acceptable. Values of p are given in the legends of the illustrations for each experiment.

Care was necessary when bees passed a test because they may have learned a cue that the experimenter did not notice or intend. Care was also necessary when bees failed a test because there may have been mutually antagonistic cues that cancelled. Failures by the bees (not significantly different from 50%) provided the most useful information, showing that they detected nothing in the test that they had learned in the training, or that inputs cancelled out.

### The setting and the illustrations

To understand the text that follows, it is essential to refer at every step to the corresponding illustration. The training patterns are shown at the top of each, followed by the set of test patterns, with scores presented as percentage correct and the number of counts. The main conclusions were that the bees detected and learned two very simple features in order of preference from the particular training patterns on hand. When they returned, they looked for the two decisive features in the test patterns as a guide to the place where they found the reward.

## RESULTS

### Polarity was not distinguished in some patterns lacking green contrast

Bees had great difficulty in distinguishing between patterns that differed in the horizontal position of an area of black or grey (Fig. 2A). The position of a black or grey area in the horizontal direction was not distinguished on a white background, and the central black/white boundary had no polarity for green or blue channels, suggesting that an additional landmark was required and all edge detectors are symmetrical, and independent of the bees’ direction of scanning.

Filling the white area with buff or yellow (Fig. 2B) did not enable bees to discriminate because there was no display of blue, and modulation detectors could not discriminate the polarity of the strong contrast at the central boundary. No polarity was detected from the outer edges because polarity required a relative position of a blue input, and the contrasts at vertical edges of buff or black on a white background were all saturated and appeared equal to the bees (Table 1).

A new group of bees could not discriminate a pattern consisting of a large buff and a large blue panel on a white background from its mirror image when there was no green contrast at the central boundary [7] (Fig. 2C). Similarly, coarse diagonal gratings (Fig. 2D) and some other patterns of two colours with no green contrast were not distinguished [8] although the areas of colour subtended 25° or more.

It has often been said that bees could not discriminate the colour of an area when there was no green contrast at its boundary, but that was no more than a restatement of a particular observation, as shown by the easy discrimination of blue contrast in spots [20] or in vertical versus horizontal gratings [8] with no green contrast. Oblique gratings with no green contrast (Fig. 2D)were not distinguished because there was equal blue and green receptor modulation and equal amount of colour on each pattern.

### A black vertical line provided the missing green contrast

A new group of bees could distinguish the polarity very well when a vertical black line 3° wide supplied a landmark in a target consisting of a buff and a blue panel with no green contrast at the central boundary (Fig. 3A). The central boundary alone provided no indication of polarity to the bees (Fig. 2C).

The bees trained on Fig. 3A distinguished the positions of the blue panels alone (Fig. 3B), but the poor response showed that at least one feature was missing or an unfamiliar item had been added. There were a number of candidates. Each target in Fig. 3B displayed an unfamiliar area of white that would stimulate both green and blue receptors, and there was unfamiliar edge along the boundary between white and blue.

When the black line was located at the outer edges of the blue panels (Fig. 3C), the apparent polarity to the bees was reversed, because the blue area remained in its retinotopic place in the training and the relative position of the black line to the blue was reversed. Bars of blue alone were insufficient (Fig. 3D).

Panels of buff and white were also not recognized (Fig. 3E). The trained bees could not distinguish between a black line on a totally buff background versus its mirror image (Fig. 3F), nor the patterns with the blue panels omitted (Fig. 3G), so there was considerable evidence that the bees had not remembered the positions of the buff panels at all.

The trained bees scored well in a test with the black line in its original position, coupled with only a thin blue bar anywhere within the area that had been blue in the training (Fig. 3H). This was the minimum pattern that gave the high score seen in the training. The contribution of the black line could only be the position of green contrast; the whole pattern was not required. Even so, the trained bees preferred the original training pattern to the skeleton pattern (Fig. 3J) because the latter displayed an unfamiliar white area. When the blue contrast at the central boundary of the training pattern was replaced by green contrast in patterns equiluminant to the blue receptors (Fig. 3K), the test score was poor but the bees still recognized the relation of the black line to the average position of the weak reflection of blue.

### The bees learned the position of blue adjacent to black

**Fig 4:**
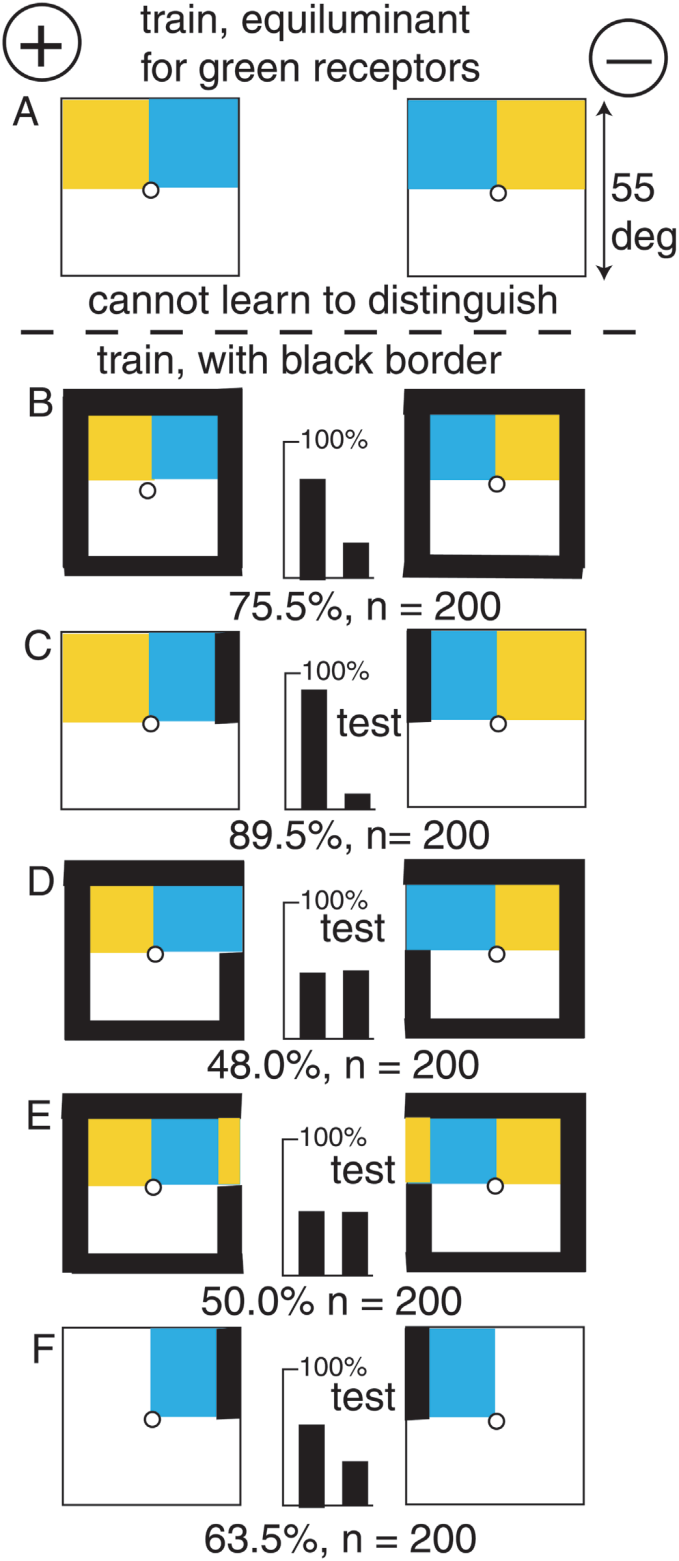
A black border enabled discrimination of equiluminant mirror images. (A) There was no discrimination with two smaller panels with no green contrast. (B) The trained bees discriminated very well when there was a surrounding black frame. (C) The trained bees distinguished with a small part of the frame. (D, E) They failed when the critical part of the frame was removed or replaced by buff. (F) The blue panel and its piece of the frame were sufficient. For scores of 58 or more and n = 200, p < 0.01 (1%), which was acceptable.

As in the previous example, a new group of bees could not discriminate between a target displaying buff and blue panels from its mirror image when there was no green contrast at the boundary between the colours (Fig. 4A). However, with a black border around the outside (Fig. 4B), a new group of bees discriminated very well, so that we can now explore what they had detected.

By removing pieces of the black margin bit by bit, it was found that the trained bees could easily discriminate when there was only a small black section adjacent to the blue panel (Fig. 4C). Moreover, they failed when only this small section was missing. Blue or buff bits of margin in the critical part of the border were equally ineffective (Fig. 4D, E). Finally, the blue panels with the small section of the black border were sufficient (Fig. 4F), but with a poor score.

### The position of black was recognized when blue was added

**Fig 5:**
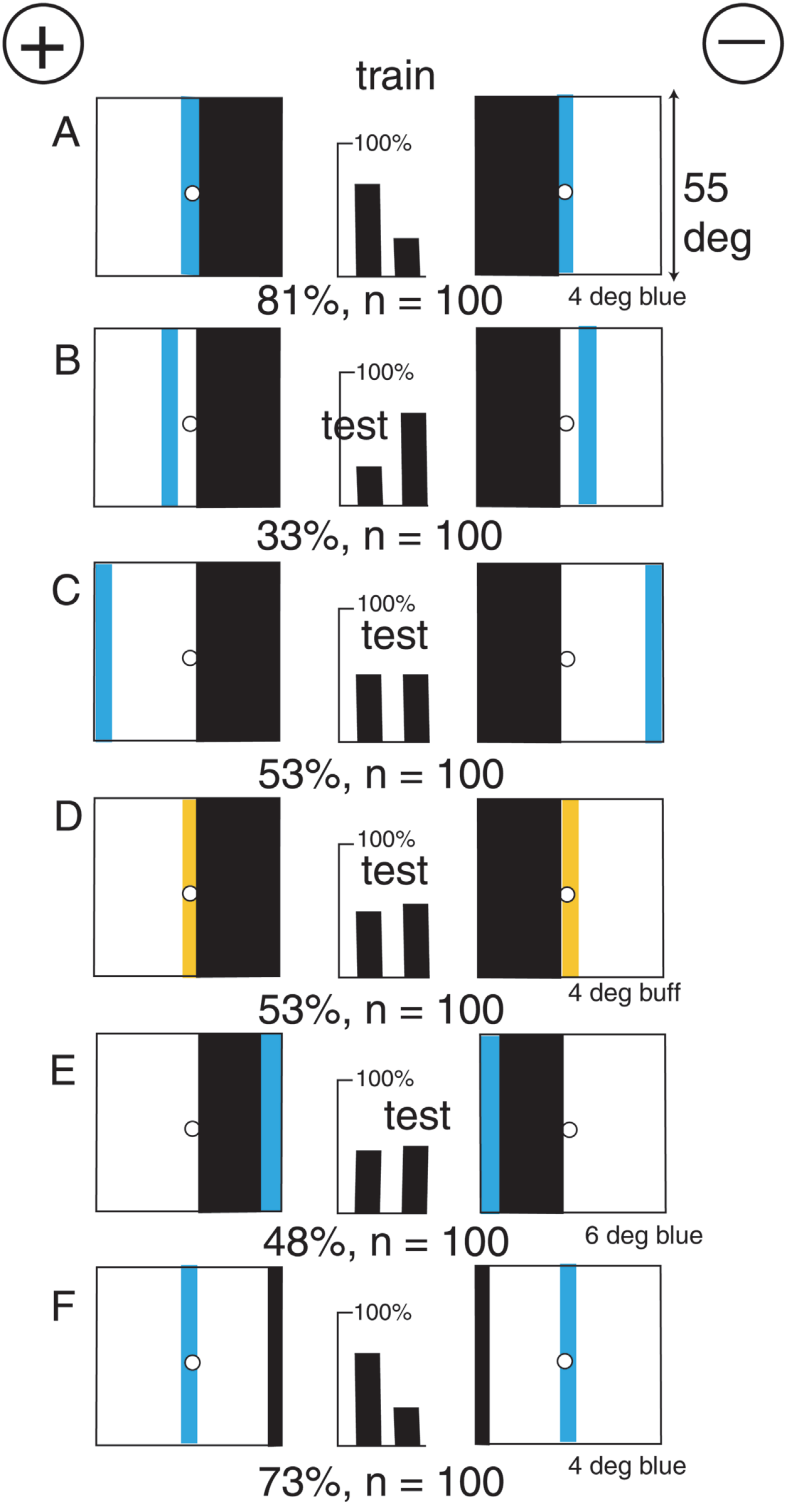
The polarity of a black/white edge was recognized when a blue bar was added. (A) Training patterns. (B) Adding a 6° white space between blue and black reversed the preference. (C) Moving the blue bar far from its position during the training spoiled the discrimination. (D) A buff bar was not effective. (E) Reversing the positions of the colours in A spoiled the response, but did not reverse the choice because the blue was not recognized outside its position in the training. (F) The trained bees discriminated patterns with blue in the centre and black at the edge contributing green contrast. For scores of 62 or more and n = 100, p < 0.02 (2%), which was acceptable.

**Fig 6:**
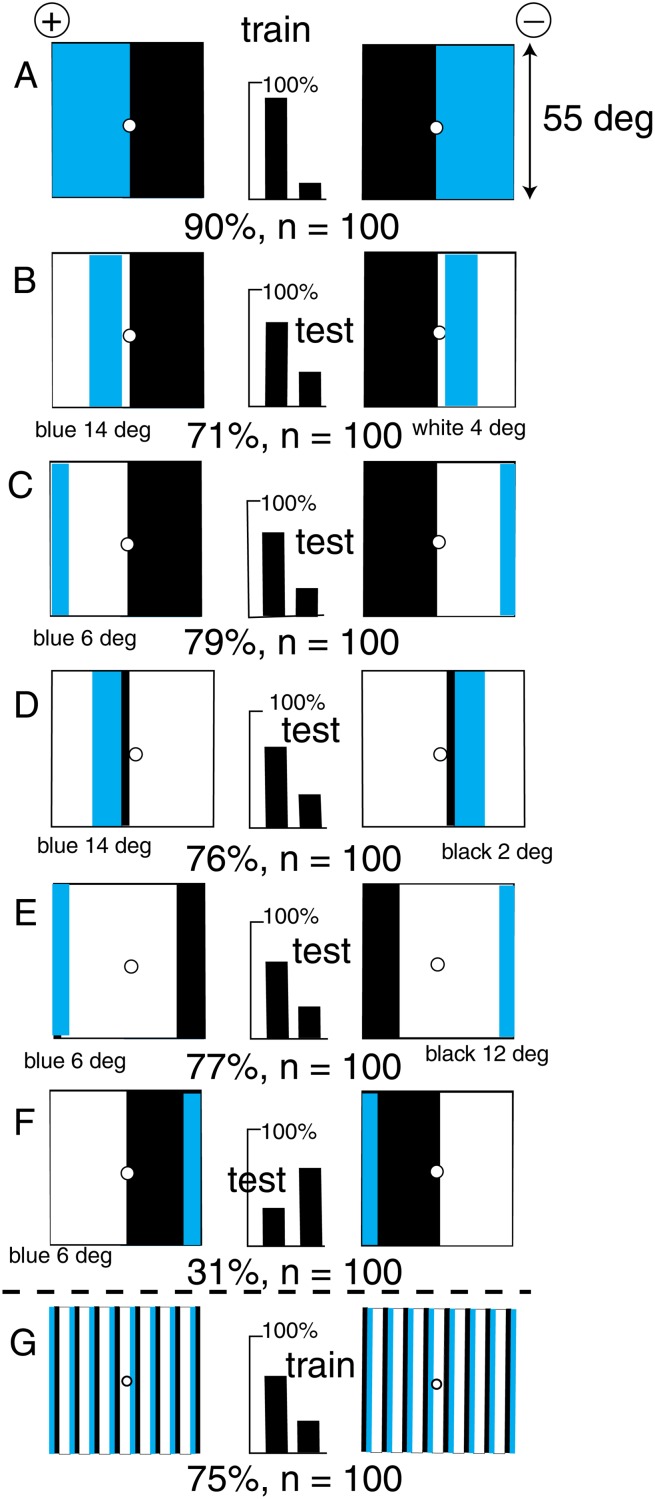
A blue and a black panel were easily distinguished from the mirror image. (A) The training patterns. (B) Introducing a white strip 4° wide reduced the score. (C, D, E) Moving the colours within their positions in the training, or reducing the widths of the panels, had little effect. (F) Putting the blue bar on the wrong side of black reversed the preference. (G) A new group of bees were trained to distinguish a vertical grating from its mirror image when there was white-blue-black polarity in each bar. For scores of 62 or more and n = 100, p < 0.02 (2%), which was acceptable.

A new group of bees failed to distinguish which side of an edge was black and which was white, with a maximum score of 58% after long training (Fig. 2A). However, the addition of a central strip of blue (Fig. 5A) enabled the bees to discriminate the patterns, although the position of the blue bar was the same on the two targets. A gap of 4° between the blue bar and the black edge was sufficient to reverse the preference (Fig. 5B) because the horizontal scanning sequence of white-blue-black was reversed (see also Fig. 6F, G). Moving the blue further spoiled the discrimination (Fig. 5C) because the blue bar had been moved away from its retinotopic position in the training. When the blue strip was changed to buff with similar stimulus to the green receptors (Table 1), the trained bees failed the test (Fig. 5D), so blue was essential. When the positions of the blue and black were reversed (Fig. 5E), the bees failed because the blue bar had been moved from its retinotopic position in the training. In a test with the blue bars alone (not illustrated) there was no recognition, showing that the black was essential. In this training pattern, the black area provided green contrast only at its distant outer edge, and the position of this black-white edge was learned relative to the retinotopic position of the blue bar (Fig. 5F).

Considering the above results (Figs. 4F, 5A), it was not surprising that a target consisting of a blue and a black panel was easily discriminated from its mirror image (Fig. 6A). The blue panel could be replaced by a blue bar anywhere in the area where blue had been displayed in the training (Fig. 6B, C, D, E), and the black panel could be reduced to a bar, with little effect on the score (Fig. 6D, E). When the blue bar was placed on the other side of the black panel, the preference reversed because the relative positions of black/white edge and blue were reversed (Fig. 6F).

A new group of bees was trained to detect the polarity in vertical gratings formed by a repeated sequence of 2° blue and 2° black bars, separated by 4° white bars (Fig. 6G). These gratings with a period of 8° were near the limit of resolution of the polarity, as shown by failures to train with similar blue-black-white gratings of smaller period. This resolution suggests that there are medium-field as well as wide-angle detectors of the spatial relation of black/white contrast and blue bar.

### In the absence of blue, green contrast was measured

**Fig 7:**
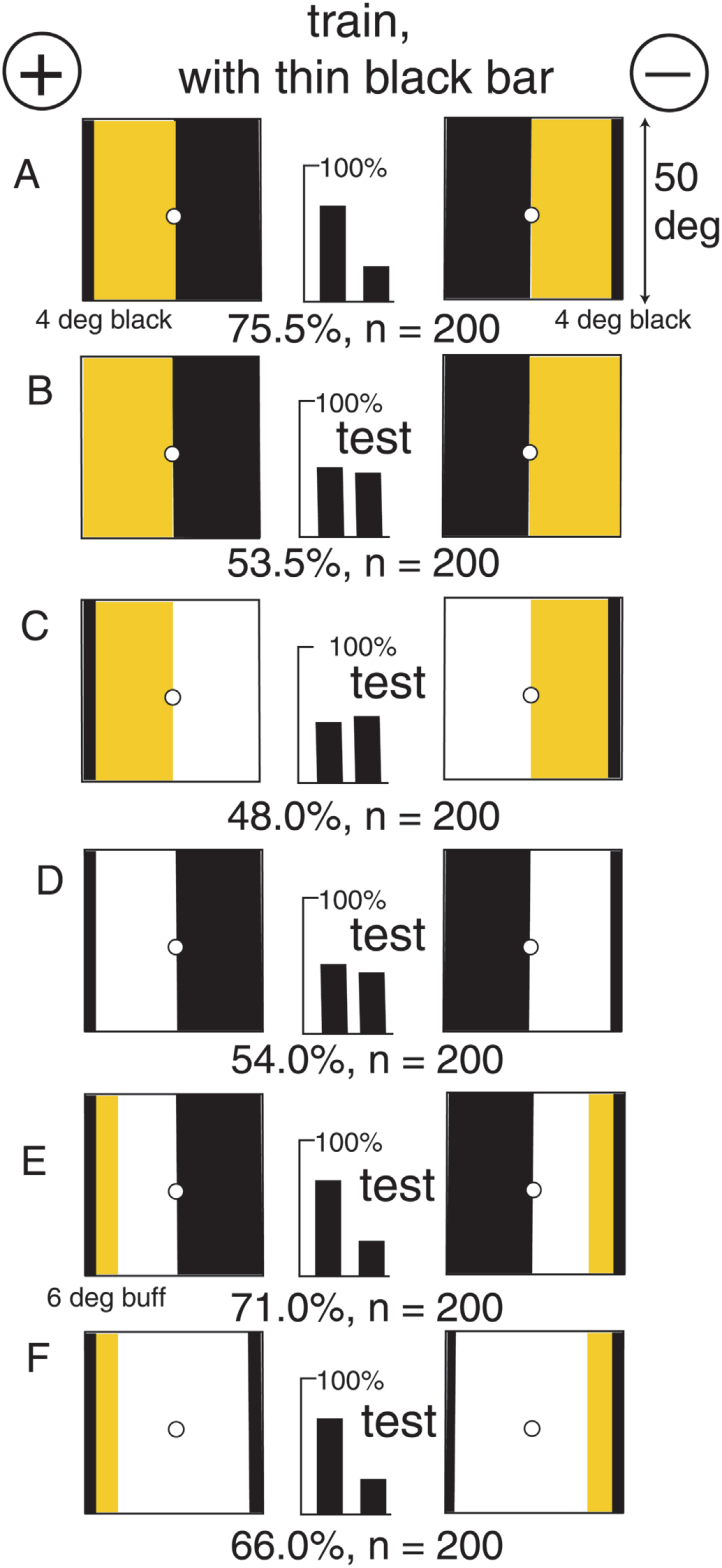
Discrimination with no difference in colour and little blue on either pattern. (A) The training patterns. (B) The black line was essential, so the bees had not learned the polarity at the central midline. (C, D) The black line with the buff or the black panel was insufficient. (E, F) A narrow strip of buff along the black line, together with the far black edge, was sufficient for discrimination. The bees had detected and learned one measure of green contrast between the buff panel and the black line and another at the outside edge of the black panel. For scores of 58 or more and n = 200, p < 0.01 (1%), which was acceptable.

A new group of bees was readily trained to discriminate a pair of patterns each consisting of a buff and a black panel when an additional 4° vertical black bar was added (Fig. 7A). Note that the four vertical outside edges were black against white and there was no blue displayed, so the cue was not obvious. The trained bees failed to discriminate when the black bar was removed (Fig. 7B) showing that something critical had been removed. Buff panels with a black edge (Fig. 7C) or a black panel and a vertical black line (Fig. 7D) were also insufficient, despite all the green and blue contrast that they displayed, showing that the critical cue was still lacking. The addition of a 6° bar of buff (Fig. 7E) brought the score back to near that in the training pattern, showing that the bees had learned a measure of the contrast at the boundary between the buff panel and the black bar and its spatial relation to the far edge of the black panel. The black panel could then be reduced to a thin bar with little effect on the score (Fig. 7F). These two components show that the bees had detected and learned the polarity from two different measures of contrast, one at the boundary between the black line and the buff panel and the other at the outer edge of the black against the white background. There was no suggestion of a tonic green receptor input from the buff panels.

The white areas in some test patterns reflected blue light, so there was abundant contrast at the internal boundaries, but the bees had not learned anything about blue in the training so they ignored the white in the tests. The result shows that when blue content was low in the training, the bees were obliged to use two separate positions and measures of contrast to determine the polarity.

### Corroboration from a different type of pattern

**Fig 8:**
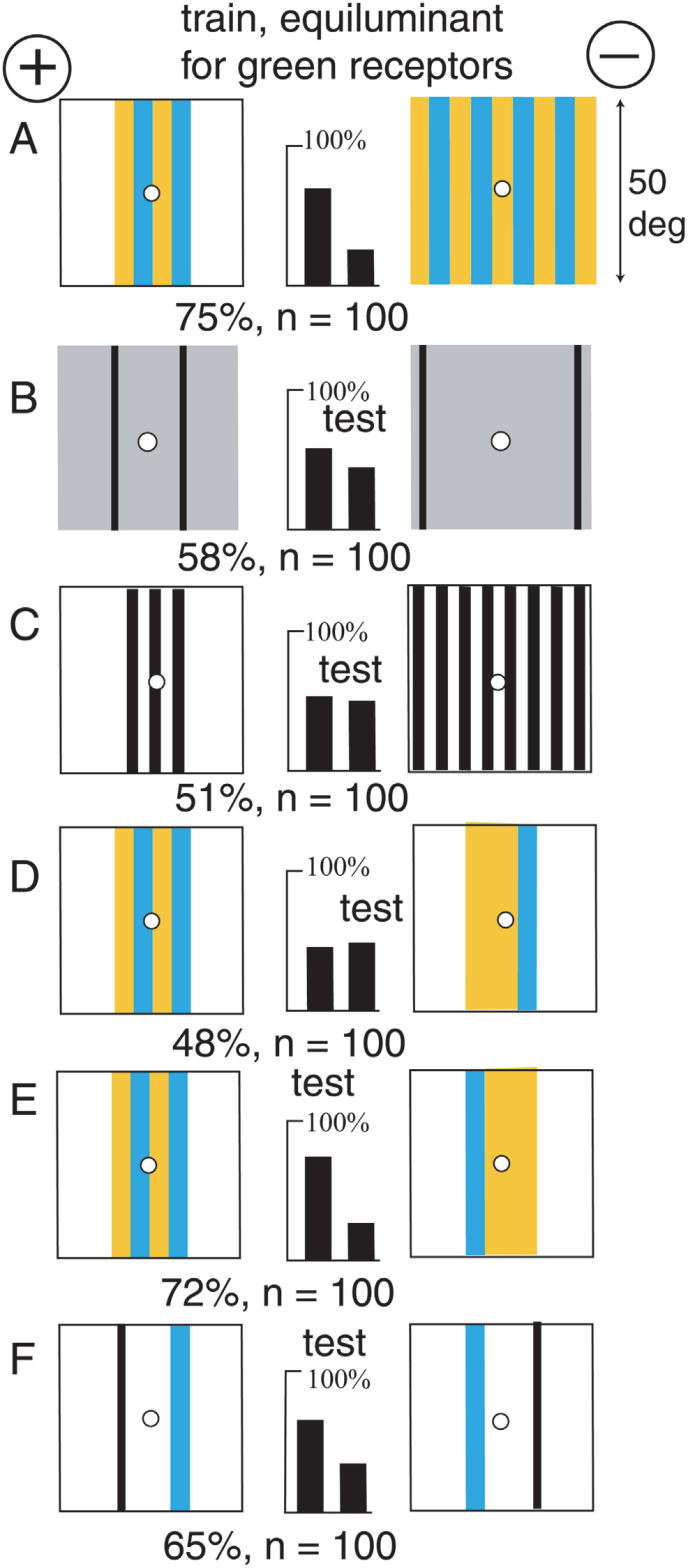
The same mechanism was found with a different type of pattern. (A) The training patterns, with different widths of gratings with no green contrast. (B, C) Two tests that failed. (D) Failure with blue on the right in both targets. (E) Success with blue on the right in one target, as in the training. (F) Test with a skeleton pattern as in the previous experiments. The bees had distinguished the polarity, not the patterns. For scores of 62 or more and n = 100, p < 0.02 (2%), which was acceptable.

While investigating how bees discriminate between grating patterns of differing width, an interesting result presented itself. A new group of bees were trained to discriminate a buff/blue pattern of four bars with no green contrast from a wider pattern of nine of the same bars (Fig. 8A). These gratings would be resolved. It was expected that the bees would learn a difference in blue contrast. Tests showed that they had not learned the width between the patterns (Fig. 8B) the amount of blue modulation in either pattern, or the difference in blue modulation (Fig. 8C).

The trained bees could not distinguish the rewarded training pattern from a wide bar of buff with a blue bar on the right side (Fig. 8D) but easily distinguished when the blue was on the left (Fig. 8E). This result showed that they had learned the position of the white/buff contrast at the left edge relative to the position of the blue bar on the right hand edge of the rewarded pattern. When tested with the skeleton pattern with the blue bar and a black bar providing green contrast at the expected locations, the bees responded correctly (Fig. 8F). Blue contrast has already been excluded (Fig. 8C). Therefore the bees used the position of green contrast at the outer edge of buff relative to the retinotopic position of blue, in preference to the available differences in width, blue modulation, and colours of the gratings.

### The detection algorithm measured angle with a retinotopic memory

**Fig 9:**
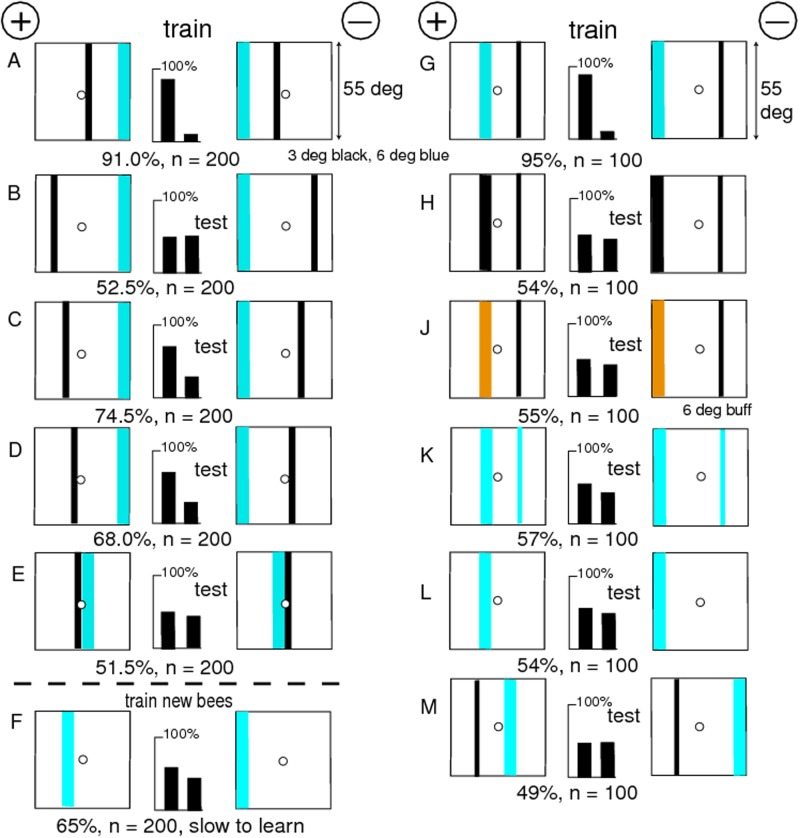
The properties of the skeleton pattern. (A). The training patterns. (B, C, D) The effect of moving the black line while the blue bar was fixed. (E) Moving the blue bar from its position in the training spoiled the discrimination. (F) Training on the blue bars alone was slow. For scores of 58 or more and n = 200, p < 0.01 (1%), which was acceptable. (G) Training with the same relative positions of blue and black but at different separations (different range) was rapid. (H, J, K) A change of colour was disastrous. (L) The blue bars alone were insufficient. (M) Turning each pattern over destroyed the discrimination, so the difference in separation of the bars was insufficient. For scores of 62 or more and n = 100, p < 0.02 (2%), which was acceptable.

A new group of bees was trained to discriminate the skeleton pattern found in all six examples (Figs. 3H, 4F, 5F, 6E, 7F, 8F) from its mirror image, and test the trained bees to reveal its properties (Fig. 9). Only a narrow blue bar was displayed in the training because it was known that a wide blue panel could be located even when alone. With the position of the blue bar held constant, the bees trained on Fig. 9A were sensitive to a displacement of the black line (Fig. 9B, C, D). Therefore the position of blue was the preferred retinotopic landmark for the bees, not the black bar.

With the blue bar close to a black line at the centre, the bees failed (Fig. 9E), because the blue had been moved from its retinotopic position in the training pattern. Bees could be trained to distinguish between two positions of the blue bar at the same side of the target (Fig. 9F), but training was slow.

Next, a group of bees were trained to discriminate between a blue bar with a black line and the same bar and line at a different separation, but with the same polarity (Fig. 9G). The skeleton pattern was, in effect, discriminated from itself at a different range. Clearly the blue bar and the black line were both essential in the tests (Fig. 9H, J, K, L). Discrimination was lost when the trained bees were tested with polarity reversed on both training patterns, but retaining the angle between bar and line (Fig. 9M). These tests show that the bees learned to measure and identify the two components, the relative positions of retinotopic blue and the green contrast at the vertical edge, and also the angle between them.

### The original pattern hides an additional variable

**Fig 10:**
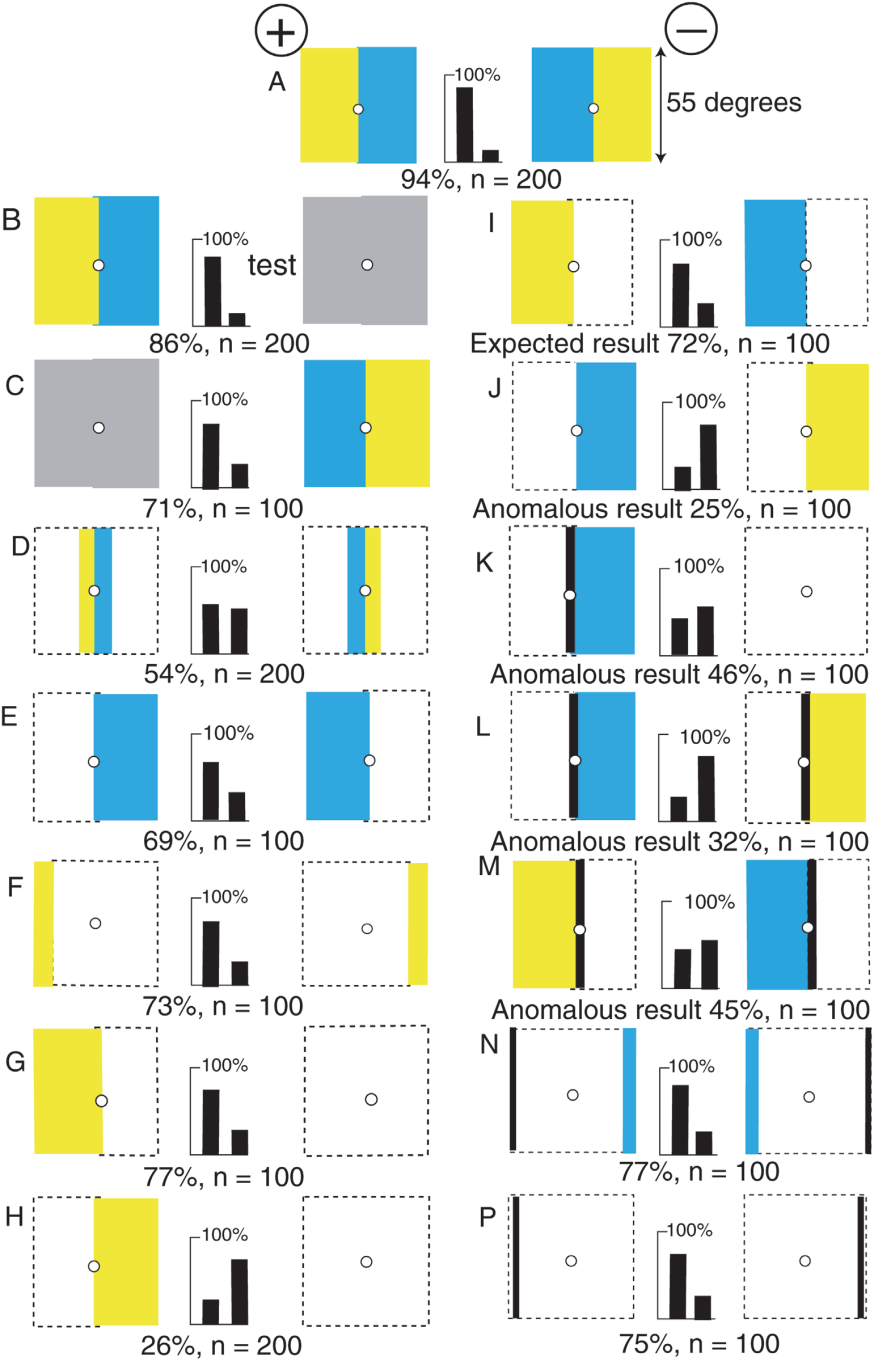
The original pair of yellow and blue training patterns revealed the effect of exposing the background white. (A) Training patterns, with blue and green contrast at the boundary and the same amounts of colour on each pattern. (B-P) Pairs of test patterns. (B, C) The trained bees distinguished between both training patterns and a plain grey target. (D) The trained bees could not discriminate the central boundary regions. (E, F, G, H) They discriminated with the blue or the yellow panels alone. (I, J) With a blue versus a yellow panel, the results were not consistent. (K, L, M) When a black line was added, preferences were lost or reversed. (N) A thin blue vertical strip, plus a vertical black line on each target, was as effective as the training pattern. (P) A single black line, with the blue content of the background, also gave a high score. For scores of 62 or more, or less than 38, and n = 100, p < 0.02 (2%), which was acceptable.

We can now return to the original example [4, 24] that displayed both tonic and phasic signals to both green and blue receptors (Fig. 1). A new group of bees rapidly learned to distinguish between a target consisting of a yellow and a blue panel versus the mirror image of the same (Fig. 10A). The trained bees strongly preferred the rewarded training pattern to plain grey (Fig. 10B), and also avoided the unrewarded target in favour of grey (Fig. 10C), showing that they had learned something from each target.

In a test that reduced the areas of colour equally (Fig. 10D), the narrower the panels, the poorer was the score, confirming that the information about polarity was not at the midline boundary.

The trained bees could distinguish the positions of the blue panels alone (Fig. 10E). A large area of blue was effective but a small area was insufficient, indicating an effect of area, not vertical edge length. The positions of the yellow panels alone were also discriminated (not illustrated), and the outer yellow edges on a white background were sufficient (Fig. 10F). A single target with one yellow panel versus a plain white target was also sufficient (Fig. 10G, H). In these tests, the white area provided a strong blue stimulus because it unexpectedly replaced retinotopic blue (see below). The performance was excellent when the test patterns were reduced to a strip of blue at one side and a thin vertical black line that generated contrast at the opposite edge (Fig. 10N). This was the simplest fully effective test pattern. The black line replaced the yellow edge, and a narrow bar of blue was effective if it was within the area where blue had been displayed in the training (Fig. 10N). The blue could be replaced by white (Fig. 10P).

At this point, it seemed that the trained bees detected the polarity of the rewarded pattern by the position of the green contrast or black line at the left outer edge (Fig. 10F, G), relative to the position of blue or white at the opposite edge (Fig. 10N), as in the previous experiments.

The results of other tests, however, showed that an additional and previously unsuspected variable contributed to the scores. A test pattern that displayed a yellow panel on the left side was consistently preferred even when the blue panel was missing (Fig. 10B, F, G, I). They avoided a yellow panel with an edge on the right side (Fig. 10C, H) unless it was combined with an unexpected area of white background that replaced a blue training panel (Fig. 10J, L). When a additional black line was added (Fig. 10K, M), the bees shifted their attention away from the contrast at the outer edges.

Blue at the right side of the test pattern was also attractive to the trained bees (Fig. 10B, E), except when the left edge of yellow was against unexpected white which displayed strong blue (Fig. 10J, L). When a white area was unexpectedly exposed by removal of a yellow panel, it caused a stronger blue stimulus than the blue itself, and so caused the reversal of preference because in that region of the eye the receptors were not adapted to the white background (Fig. 10J, L).

### A black background makes a more elegant experiment

**Fig 11:**
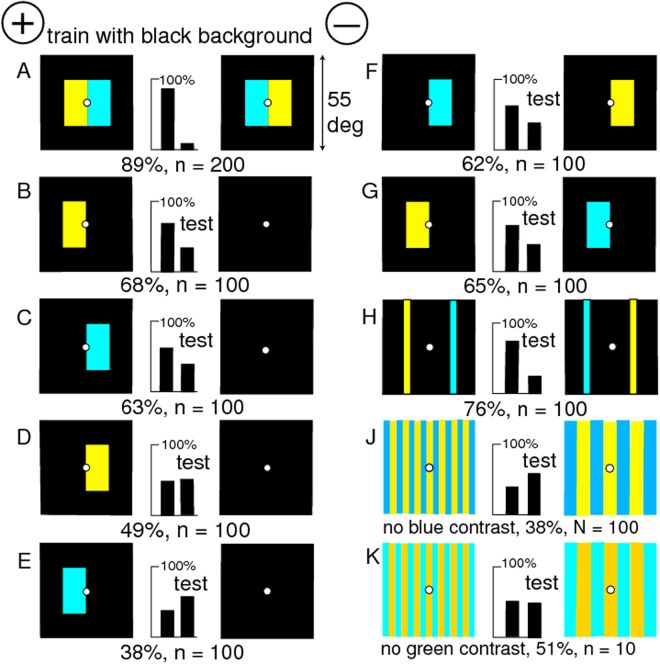
Discrimination with a black background; removing a coloured panel exposed no unexpected white. (A) Training patterns. (B-E) Tests with a single panel versus black, or (F-G) with one panel of each colour on each target, show that yellow was not learned on the unrewarded target and memory was retinotopic. (H) Thin yellow and blue bars served as well as the training patterns. (J, K) The trained bees preferred the greater period in gratings with no blue contrast, but showed no preference with gratings with no green contrast. For scores of 62 or more, or 38 or less, and n = 100 in all tests, p < 0.02 (2%), which was acceptable.

As corroboration of the effect of unexpected exposure of white background, a new group of bees was trained with yellow/blue patterns on a black background (Fig. 11A). When tested versus a plain black target, they recognized with difficulty the two separate halves of the rewarded pattern (Fig. 11B, C) but not the yellow panel of the unrewarded target (Fig. 11D; compare Fig. 10G, H). They avoided the blue panel from the unrewarded target (Fig. 11E). These four tests show that the memory of a blue area was retinotopic and that yellow and blue were not treated in the same way. When tested with the two right or the two left halves of the training targets (Fig. 11F, G), the trained bees responded as would be inferred from earlier tests, with no anomalous result. They responded very well to black targets with a thin band of yellow and one of blue (Fig. 11H), but failed with bands of yellow or blue alone (not illustrated). When tested with two gratings of hemp and ultramarine, with no blue contrast (Fig. 11J), they preferred the one generating the most green modulation, but they showed no preference with gratings differing in blue contrast but equiluminant for green (Fig. 11K). The edge of the yellow panel was a source of green contrast but there was no evidence that the bees remembered yellow colour or blue contrast. These results are consistent with the previous findings; bees learned the retinotopic positions of blue and the green contrast at the outside edge of one yellow panel. There was no anomalous result caused by the unexpected exposure of a white background.

## DISCUSSION

The bees avoided the question of colour vision by recognition of the spatial relation between two different inputs. Humans call this polarity but bees measure and locate two inputs, then recognize the same inputs and the angle between when they returned for more reward. In every other example of bee vision of black-white patterns analyzed in the same way, the bees learned a similar coincidence of relatively simple inputs with no suggestion of vision of pattern or trichromatic colour [12, 17, 20]. In all the experiments, black necessarily generated the maximum green and blue contrast at its boundary with any other colour, and the bees were quick to use this strong signal. Patterns on a background of white appeared to display no differences in contrast at their outer borders (Figs. 2 and 4A), because the contrasts were saturated, or because the apparatus was painted white inside so that the bees were adapted to white.

These results, and especially the failure to distinguish some coloured patterns (Fig. 2), introduced a doubt that the bees could detect the lay-out of patterns. In fact, when we consider the size of the coloured areas in Fig. 2, it is hard to believe that bees discriminate colours. There was no evidence that the bees were interested in the ratios of the tonic receptor responses or in chromatic contrast. They preferred to measure the monochromatic blue input and made use of a buff, yellow or black vertical line or edge to quantify the green contrast. The preferred retinotopic positions of the green contrast and blue were always far apart on the target, optimum for triangulation. However, we cannot rule out the possibility that the bees also discriminate colour in other situations.

The results revealed the kind of system in the recognition of coloured pattern. In every experiment bees detected the same few discrete inputs, so the memory channels were limited to monochromatic blue and receptor modulation. Two out of three variables (blue content and two modulations) were measured and located retinotopically, and the angle between them measured, as if for triangulation of their geometrical arrangement.

In previous work with black and white training patterns the bees also learned simple features. The orientations of orthogonal edges were summed in local regions so that pattern was destroyed. It was abundantly demonstrated that pattern and shape were not re-assembled [9–11, 20]. We now find that they did not learn the lay-out of colour. The asymmetry at a boundary between two colours did not indicate polarity. White, which excited all the receptors, played little part as a background because the bees adapted to it, but had a large effect when a white background was suddenly locally exposed (Fig. 10). Long before this analysis, Friedlaender [24] had used training and test patterns similar to my Fig. 10E, G, H, I and J. Although she used small patterns 3 cm by 3 cm on a vertical white background, and the criterion was the bee’s landing on a central reward hole, her results were similar to mine (Fig. 10J), but there was no explanation of the anomalous result at that time.

The results said little about the brain or behaviour of bees, but they revealed a great deal about the inputs into the bees’ eyes. The detection of a coincidence of two inputs was suitable for a system with a very wide field of view that scanned in the horizontal plane. It made use of both tonic and phasic signals, wide and narrow fields, and the retinotopic structure of the eye. The two inputs could be widely separated spatially and they matched the large field sizes of the blue detectors and small field sizes of the green modulation detectors. In the natural panorama, polarity would act as a directional signpost. So far, all of the first stages of visual recognition appeared to be rapid, automatic and limited to these few types of feature detectors.
